# Pathogenesis of Behçet's Syndrome: Genetic, Environmental and Immunological Factors

**DOI:** 10.3389/fmed.2021.713052

**Published:** 2021-10-08

**Authors:** Irene Mattioli, Alessandra Bettiol, Güher Saruhan-Direskeneli, Haner Direskeneli, Giacomo Emmi

**Affiliations:** ^1^Department of Experimental and Clinical Medicine, University of Firenze, Firenze, Italy; ^2^Department of Physiology, Istanbul Medical Faculty, Istanbul University, Istanbul, Turkey; ^3^Department of Internal Medicine, Division of Rheumatology, School of Medicine, Marmara University, Istanbul, Turkey

**Keywords:** Behçet's syndrome, pathogenesis, HLA-B^*^51, neutrophils, epigenetic, microbiome

## Abstract

Behçet's syndrome (BS) is a rare systemic vasculitis, characterized by a wide range of different clinical involvements and unpredictable phases of recurrence and remission. BS can be described as a multifactorial disease with an incompletely known etiopathogenesis; in fact, though presenting some peculiar features, such as its typical geographic distribution and the strong association with the well-known genetic predisposing factor HLA-B^*^51, the cause behind the onset and progression of the disease remains currently not fully understood. Besides genetic HLA and non-HLA predisposing associations and epigenetic influence, environmental factors also play an important role in the pathogenesis of the disease, and among these, infectious agents (both bacterial and viral) and specific microbiome alterations are considered of particular relevance in BS pathogenesis. BS has been included for decades among autoimmune diseases, in light of evidence showing T- and B-cell aberrant responses. However, because of recurrent mucocutaneous lesions and episodes of inflammation without antigen-specific T-cell or autoantibody responses, BS has also been classified among autoinflammatory disorders. Nevertheless, differently from autoinflammatory diseases, BS mildly responds to therapies targeting IL-1, its onset is not usually in childhood, and has high neutrophilic vasculitic involvement. Finally, given the association with HLA class I alleles, similar to spondyloarthropathies, the concept of BS as a major histocompatibility complex (MHC) I -opathy has been introduced. Understanding the complex etiopathogenesis of BS is essential to identify modifiable risk factors of BS occurrence or exacerbation and to develop targeted therapies. This review summarizes current evidence on the main genetic, environmental and immunological factors contributing to BS development.

## Introduction

Behçet syndrome (BS) is a rare systemic vasculitis, characterized by a wide range of different clinical involvements, which have unpredictable phases of recurrence and remission (1). Moreover, the different clinical manifestations may present alone, or co-exist in the same patient (2, 3).

BS can be described as a multifactorial disease with an incompletely known etiopathogenesis, and unique geographic distribution suggests that both genetic and environmental susceptibility factors might be involved. Indeed, the global pool prevalence of BS has been estimated around 10.3 (95% CI: 6.1–7.7)/100.000 people, but it is considerably higher in the countries along the ancient Silk Route (e.g., Turkey and Iran) (4).

BS has been included for decades among autoimmune diseases, in light of evidence showing T- and B-cell responses to heat-shock proteins (HSP), endothelial cells, enolase and retinal S antigen (5). However, there are some features that do not support the autoimmune nature of the disease, such as lack of anti-nuclear antibodies, female prevalence or increased risk of autoimmunity (6).

Because of its symptomatology including recurrent non-scarring mucocutaneous lesions and non-deforming arthritis, and episodes of inflammation without antigen-specific T-cell or autoantibody response, elevated neutrophil activation, and increased levels of certain proinflammatory cytokines, such as interleukin (IL)-1, BS has also been classified among autoinflammatory disorders (7, 8).

However, unlike other autoinflammatory diseases, BS mildly responds to therapies that specifically target IL-1, the first symptoms usually begin after puberty rather than in childhood, and there is generally high vasculitic involvement, which is quite rare in autoinflammatory diseases. According to the Chapel Hill classification revised in 2012, it is now counted among systemic vasculitis with variable caliber vessel involvement (9).

In recent years, clinical studies have shed light on a possible similarity with spondyloarthropathies (SpA), introducing the concept of BS as a human major histocompatibility complex (MHC)-I-opathy (10). This similarity is supported by the association of specific human leukocyte antigen (HLA) class I groups, namely HLA-B^*^51 and HLA-B^*^27, and the development of BS and SpA, respectively (11).

**Figure 1:**
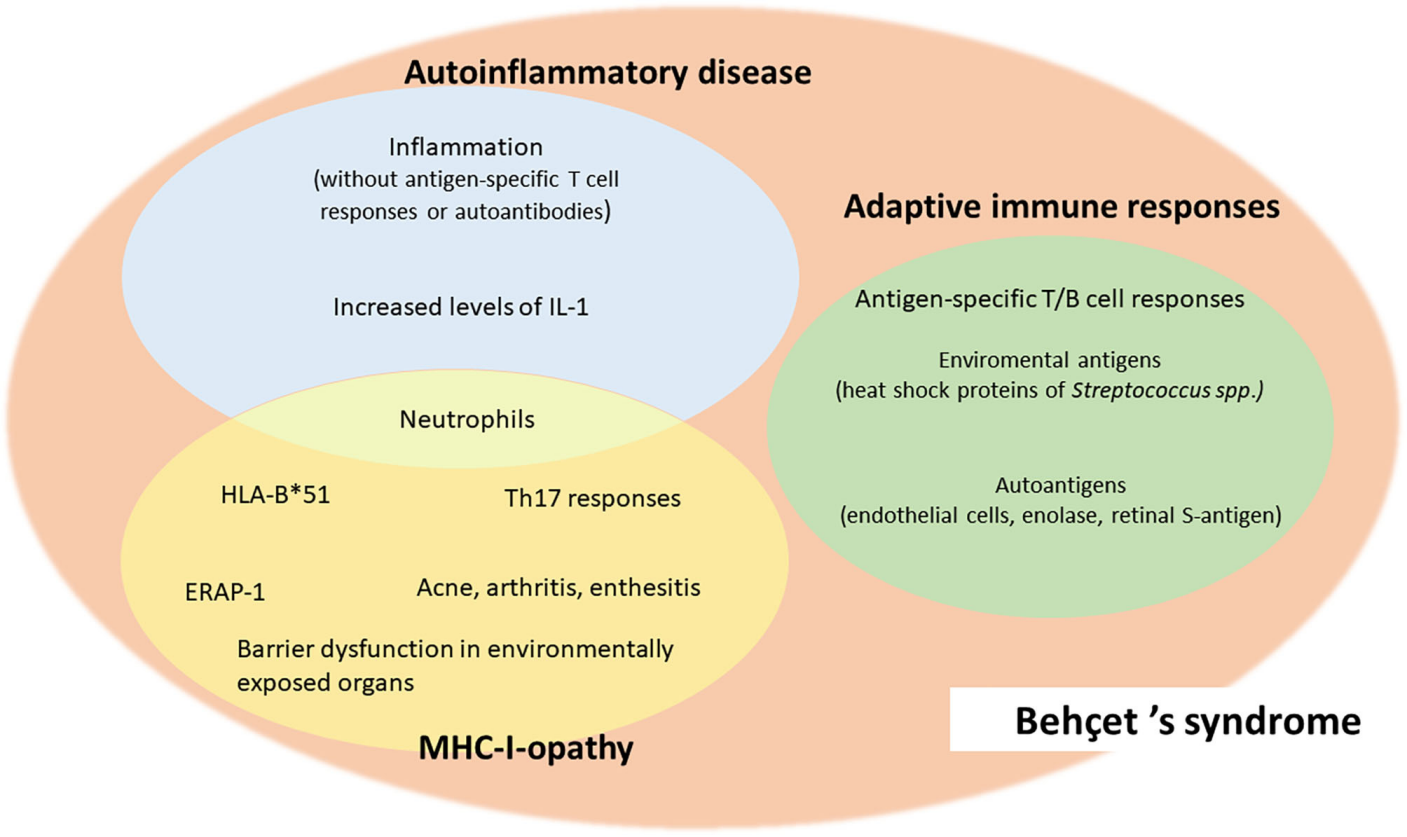
Common features of Behçet's syndrome and autoimmune diseases, autoinflammatory diseases and MHC-I-opathies.

Although to date BS cannot be unambiguously classified as an autoimmune or an autoinflammatory disorder, a systemic vasculitis or a disease belonging to the MHC-I-opathies, but possesses common features with all these groups (Figure 1), it is increasingly important to categorize the disease because it helps understanding the underlying pathogenetic mechanisms, thus choosing the most appropriate therapeutic strategy.

It is now known that the complex etiopathogenesis of BS involves more than one pathogenetic pathway, namely (12):

(1) Genetic and epigenetic factors, including geographic distribution, the association with HLA and non-HLA genes, and micro-RNA (miRNA) polymorphisms.
(2) Environmental etiology, including infections, microbiome, and additional triggering factors.
(3) Immunological pathways, including neutrophils and other immune-mediated mechanisms of damage.

## Genetic and Epigenetic Etiology

### Genetic Factors

Different environmental factors can lead to epigenetic changes that may account for an increased incidence of the disease in some geographic areas. Similarly, the geographic distribution of BS has always implicated a genetic predisposition to the disease. The HLA class I antigen, HLA-B^*^51, has been identified as the predominant genetic susceptibility factor underlying BS in many populations, especially along the ancient Silk Road that ran from East Asia to the Middle East and Mediterranean basin (2, 13).

However, the frequency of HLA-B^*^51 in some ethnic groups (e.g., Italians and Portuguese) is similar to that in Silk Road areas, despite a much lower prevalence of BS (14), proposing that unknown environmental factors may interact with HLA-B^*^51 alleles, driving BS development.

A meta-analysis of data from 78 independent studies has shown that subjects presenting the HLA-B^*^51/B^*^5 had a 5-fold increased risk of BS compared with non-carriers (15). Although HLA-B^*^51 is the known genetic factor most closely associated with BS, it accounts for <20% of the genetic risk (16). Some other genes in HLA region such as Cw^*^1602 and A^*^26 are also found to be associated with BS (17).

In addition to the MHC class I region, several non-HLA genes are associated with BS. Common variants in *IL10, IL23R*-*IL12RB2* loci and signal traducer and activator of transcription (STAT)-4 predispose individuals to BS (18). Expression studies have shown that disease-associated *IL10* variants are correlated with reduced expression of this anti-inflammatory cytokine, which can lead to a susceptible inflammatory state, increasing BS susceptibility (16).

Other susceptibility genes recently identified by GWAS are *ERAP1* (encoding for endoplasmic reticulum aminopeptidase 1, implicated in antigen presentation) and CCR1-CCR3, encoding for the chemokine receptor family, which play an important role in inflammatory cell recruitment and activation (19).

Another study has recently shown that non-synonymous variants (NSVs) of a gene involved in innate immune response (i.e., Toll-like receptor 4, TLR4) are associated with BS; also, the familial Mediterranean fever (*MEFV*) gene Met694Val mutation seems to confer BS risk in the Turkish population. Thus, the disease-associated NSVs in TLR4 and MEFV genes support the hypothesis of the correlation between innate immune response and bacterial sensing mechanisms in the pathogenesis of BS (19).

The NF-κB pathway is also increasingly implicated in the pathogenesis of BS (20). In this regard, it has been shown that the inflammatory features of BS might be associated with NF-κB hyperactivation in immune cells. Specifically, mutations in the *TNFAIP3* gene encoding for the regulatory protein A20, a potent inhibitor of the NF-κB signaling pathway, have been shown to increase the risk of BS development. Indeed, A20 is a 790 amino acid protein encoded by the *TNFAIP3* gene on chromosome 6 (20), which negatively regulate inflammation and immunity. Loss-of-function mutation in the *TNFAIP3* gene leads to haploinsufficiency of A20 a monogenic auto-inflammatory/auto-immune disease, with Behçet-like features (21–23).

### Epigenetic Factors

Epigenetics, namely the study of inheritable variations in gene expression, which do not involve alterations in the DNA sequence, is becoming increasingly available in BS (24). Differential methylation of genes associated with cytoskeleton remodeling and cell adhesion in CD4+ T cells and CD14+ monocytes has been identified in BS patients (25). Significantly, these alterations return to normal after the use of colchicine, an alkaloid with a well-known anti-inflammatory and anti-neutrophilic activity, widely used for different BS manifestations. Colchicine inhibits mitosis, also by interfering with genes implicated in microtubule structure (KIFA2 and TPPP), suggesting that methylation patterns could be useful as biomarkers or therapeutic targets which need further validation (26).

In addition, some evidence suggests a role of specific miRNA polymorphisms, another major form of epigenetic influence that may be involved in the pathogenesis of BS. In particular, altered expression of miRNA-155, miRNA-638, and miRNA-4488 has been described in BS (27, 28). In addition, the homozygous CC genotype and the C allele of the pre-miRNA-146a rs2910164 polymorphism were found to be protective against BS, whereas the rs3746444 and rs28362491 polymorphisms in miRNA-499 and the NF?B1 promoter were found to be involved in the genetic susceptibility of BS (29). Ubiquitination is another epigenetic factor that might influence the susceptibility to BS (30).

To sum up, epigenetic influences deserve attention in BS, as they may be instrumental in explaining the geographic and environmental differences that are prominent in this condition.

## Environmental Etiology

### Infectious Factors and Microbiome

Infectious agents have long been proposed as triggering factors in BS development (31). Antigens from viruses such as herpes simplex virus (HSV)-1 or bacteria belonging to Streptococcus species such as Streptococcus sanguinis have been suspected to have high homologies with human proteins such as human HSP, leading to a cross-reactive immune response in genetically predisposed individuals (32).

Considering that BS usually starts from the oral mucosa and that oral ulcers are almost pathognomonic in BS, especially after dental procedures, it has been hypothesized that oral microbial flora may be implicated in the pathogenesis of the disease (33). Indeed, higher levels of various strains of streptococci were found in the oral mucosa of BS patients (29).

Moreover, HSP-specific T cells are known to mediate tissue damage either through the production of Th1 cytokines, such as IFN-γ and TNF-α, or through activation of cell-mediated cytotoxicity (34, 35). Some studies have also demonstrated the association between chronic Chlamydia pneumoniae infection and the development of BS (36).

In addition to the aforementioned role of bacterial infections in the development and course of BS, viral infections, in particular HSV, have also been identified as possible triggers. Studies in Behçet-like mouse models and in BS patients demonstrated elevated levels of serum IgA and IgG anti-HSV antibodies against the UL48 protein of HSV (37). Moreover, the presence of HSV-1 DNA has been demonstrated in peripheral blood polymorphonuclear cells and in oral and genital ulcers (38).

Beyond infectious agents, the endogenous microbiota has also been suggested to be potentially involved in BS pathogenesis. Namely, a poorer and dysbiotic salivary and fecal microbial community has been described in BS compared to healthy controls (39, 40). Indeed, oral flora had less diversity, with *Haemophilus parainfluenzae* as the most overabundant species, while *Alloprevotella rava* and genus *Leptotrichia* species are depleted (40). Similarly, fecal samples from BS patients have a lower bacterial diversity in terms of *Roseburia, Subdoligranulum, Megamonas, Prevotella*, and butyrate-producing bacteria *Clostridum spp*. and methanogens (41), whereas a higher abundance of *Bifidobacterium, Eggerthella, Bilophilia spp*. and opportunistic pathogens has been described (42). Reduced microbial diversity has been associated with lower butyrate production, one of the most representative short-chain fatty acids (SCFA), able to mediate T regulatory cell (Tregs)and activation of immunopathological T-effector responses (39, 43).

Supporting the key role of butyrate in BS pathogenesis, it has recently been shown that two different butyrate-enriched diets are able to improve disease activity, while also modulating the blood redox and prothrombotic status, which is impaired in BS (44).

### Additional Triggering Factors

Beside infectious and microbiome, a wide spectrum of additional triggering factors may contribute to the etiopathogenesis of BS (31). Increasing evidence suggests a role for environmental trigger factors in the development of BS in patients with genetic susceptibility.

An association between oral ulcer recurrences and an external event, such as stress, fatigue, and food intake are reported in 78.3% of patients with BS (45). The results of these and other studies on psychological factors related to BS suggest that these factors may be considered as main triggers of oral ulcer recurrence in these patients (46, 47).

Next to psychological factors, some foods have been identified as triggers for the development of BS-related oral ulcers. Specifically, nuts, cheese, citrus fruits, pineapple, and strawberries have been reported as triggers for the development of oral ulcers (48), whereas eggplant, walnut and tomato are implicated in another series (45). Regarding patient-related factors, hormone levels have been linked to disease activity. In particular, menstruation has been described as a trigger of skin and mucosal lesions in women with BS (49), whereas testosterone has been associated with neutrophil activation in men with BS (50).

In contrast, although smoking habit reduces oral mucocutaneous inflammation in BS, it is responsible for increased atherosclerosis and increased risk of vascular events, particularly in men with BS carrying glutathione S-transferase gene polymorphisms (51).

Finally, the presence of atopic disorders, such as respiratory, skin, or food allergies, have also been associated with a lower risk of BS (31). This is probably due to the balance between pro-inflammatory Th1- and Th17-associated responses typical of BS and Th2-associated IL-4 and IL-13 responses typical of allergies (52).

## Immunological Etiology

### Neutrophil Mediated Mechanism of Damage

Neutrophils are known to play a key role in the etiopathogenesis of BS, which has traditionally been considered a neutrophilic vasculitis (53). Hyperactivated neutrophils in perivascular infiltration and tissue injury have been described in BS patients (54). These granulocytes have shown increased phagocytosis and superoxide production, potentially contributing to clot formation by fibrinogen oxidation (55). Indeed, it has been recently shown that neutrophil activation promotes fibrinogen oxidation and thrombosis formation in BS. Specifically, these findings suggest that altered fibrinogen structure and fibrinogen function are related to neutrophil activation and production of enhanced ROS, which are mainly derived from neutrophil NADPH oxidases (55). In addition to the role of ROS, other mechanisms implicated in typical thrombo-inflammation are also documented, including the release of neutrophil extracellular traps (NETs) (56). Under inflammatory or infectious conditions, neutrophils are able to generate NETs through a distinct cell death process called NETosis (57). NETs consist of extruded cell-free DNA (CfDNA) decorated with histones and granular components that include antimicrobial peptides and proteases. Molecular pathways leading to NETosis include calcium mobilization, generation of ROS, nuclear delobulation involving the enzymatic activities of myeloperoxidase (MPO) and neutrophil elastase, and chromatin modification through citrullination of histones by peptidyl arginine deiminase (PAD)-4 (58, 59).

Notably, among the major producers of NETs is a specific subtype of neutrophils with proinflammatory activity, namely low-density granulocytes (LDGs) that are widely involved in the pathogenesis of cardiovascular manifestations associated with various autoimmune and autoinflammatory disorders (60, 61). Moreover, NETs are now recognized as a key factor in the initiation and progression of thrombosis in pathological conditions such as deep vein thrombosis in mice and humans, but also in arterial diseases such as stroke and myocardial infarction (62, 63).

BS patients have been shown to be more prone to NETosis than healthy controls (64, 65), and the role of NETs in BS has been mainly implicated in the pathogenesis of vascular manifestations (58, 66, 67).

However, considering that in BS a dense neutrophilic infiltration can be found not only at the vascular level, but also at the cutaneous, articular, ocular, intestinal, and neurological locations, it is possible to assume that neutrophils and neutrophil-derived products might also be involved in the pathogenesis of non-vascular BS involvement (68).

### Other Immunological Mechanisms

In addition to neutrophils, natural killer (NK) cells are also known to play a role in BS. NK cells are the main components of innate immunity and not only play a cytotoxic role in infected cells, but also regulate the function of other immune cells, including dendritic cells (DCs) and T lymphocytes (69). Among their abilities, NK cells mediate the release of cytokines implicated in the pacing of CD4+T cells toward differentiation into Th1 cells (70), the presence of which is associated with BS activity. Therefore, overproduction of inflammatory cytokines by innate immune cells such as NK or other immune-cells (e.g., macrophages and DC) may cause a higher production of adaptive Th1- and Th17-related cytokines. Additionally, a decreased regulation by Tregs and a Th1/Th17 cytokine polarization of CD4+ T cells is a consistent finding in disease lesions and peripheral blood from BS patients, with IFN-γ, TNF-α, IL-8 and IL-17 levels correlating with BS activity (71).

Specifically, at the level of antigen-processing cells (APCs), a series of molecules such as ERAP1 assists to the exposure of microbial antigens on the surface of the same cells via MHC class I and class II molecules. The linking of activated CD4+ T lymphocytes to APCs presenting these antigens on MHC molecules is responsible for up-regulation of Th17 and Th1 lymphocytes and down-regulation of Treg lymphocytes (72).

Cytotoxic Th1 and Th17 cells are directly involved in mucosal damage in the early stages of intestinal BS involvement (73). In addition, increased levels of IL-17, IL-23 and IFN-γ have been described in patients with active ocular, mucocutaneous, and joint involvement (74). In turn, IL-17 and IFN-γ secreted by lymphocytes from BS patients have been shown to induce innate responses, late adaptive immunity, and neutrophil infiltration (75). These immune features are shared between different spondyloarthropathies and support the concept of MHC-I-opathies (10).

Upregulation of different Th subtypes may also be linked to upregulated Janus kinase (JAK)/STAT pathway in both CD4+ T cells and CD14+ monocytes in BS (76). Recently IFN-γ receptor-1 (*IFNGR1*) gene has been shown as a susceptibility locus for BS in a large multi-ethnic GWAS study (77). *IFNGR1* encodes the binding subunit, α chain, of the IFN-γ receptor and the binding of IFN-γ stimulates the activation of JAK-STAT signaling pathway. BS-associated variant leads to higher levels of expression of the receptor after stimulation *in vitro*, suggesting a functional effect in BS pathogenesis.

Furthermore, CD8 + T cells also play a significant role in BS pathogenesis. Indeed, some studies showed a significant association between CD8+ T cells and disease activity in BS patients, thus suggesting their potential critical role in BS recurrence (78, 79).

Finally, along with CD4+ and CD8+ T cells, circulating γδ T cells in BS patients have also been correlated with disease status. γδT lymphocytes also play an important role in the regulation of the autoimmune response. γδT cells are a minor population of T cells present in ~0.5–5% of total blood, which express the γ and δ chains of the T cell receptor (TCR), providing an essential contribution to many types of immune response. Among their pleiotropic effector functions, they are able to recognize qualitatively distinct antigens, protect multiple defined anatomical sites, and are capable of mediating and modulating responses to specific pathogens (80).

γδT cells have been identified in BS inflammatory lesions and have been suggested to be contributory to the induction and/or maintenance of the proinflammatory environment characteristic of the disease (81).

## Discussion

Understanding the pathogenesis of BS is a critical step in the development of novel and effective therapies. A genetic predisposition (HLA and non-HLA-driven), with an abnormal innate hyperinflammatory response and neutrophil hyperactivity are well-known hallmarks of the disorder.

On this susceptible background, environmental and microbial risk factors can trigger inflammation of innate origin, which can subsequently be sustained by adaptive immune responses.

However, to date, the etiopathogenesis of the disease remains poorly understood. Recent findings of monogenic diseases sharing features with BS strongly suggest an important role for dysregulated innate immune activation due to mutations in autoinflammatory cascades such as NF- kB pathway in familial and polygenic BS cases. Activation of JAK/STAT pathway associated with *IFNGR1* polymorphism and pro-inflammatory cytokine milieu (IL-6, IL-17) leading to Th1/Th17 activation is also implicated.

Moreover, T cells also play a crucial role, especially CD8+ T cells, as they seem to be correlated with disease status and therefore representing a potential therapeutic target for BS.

**Figure 2:**
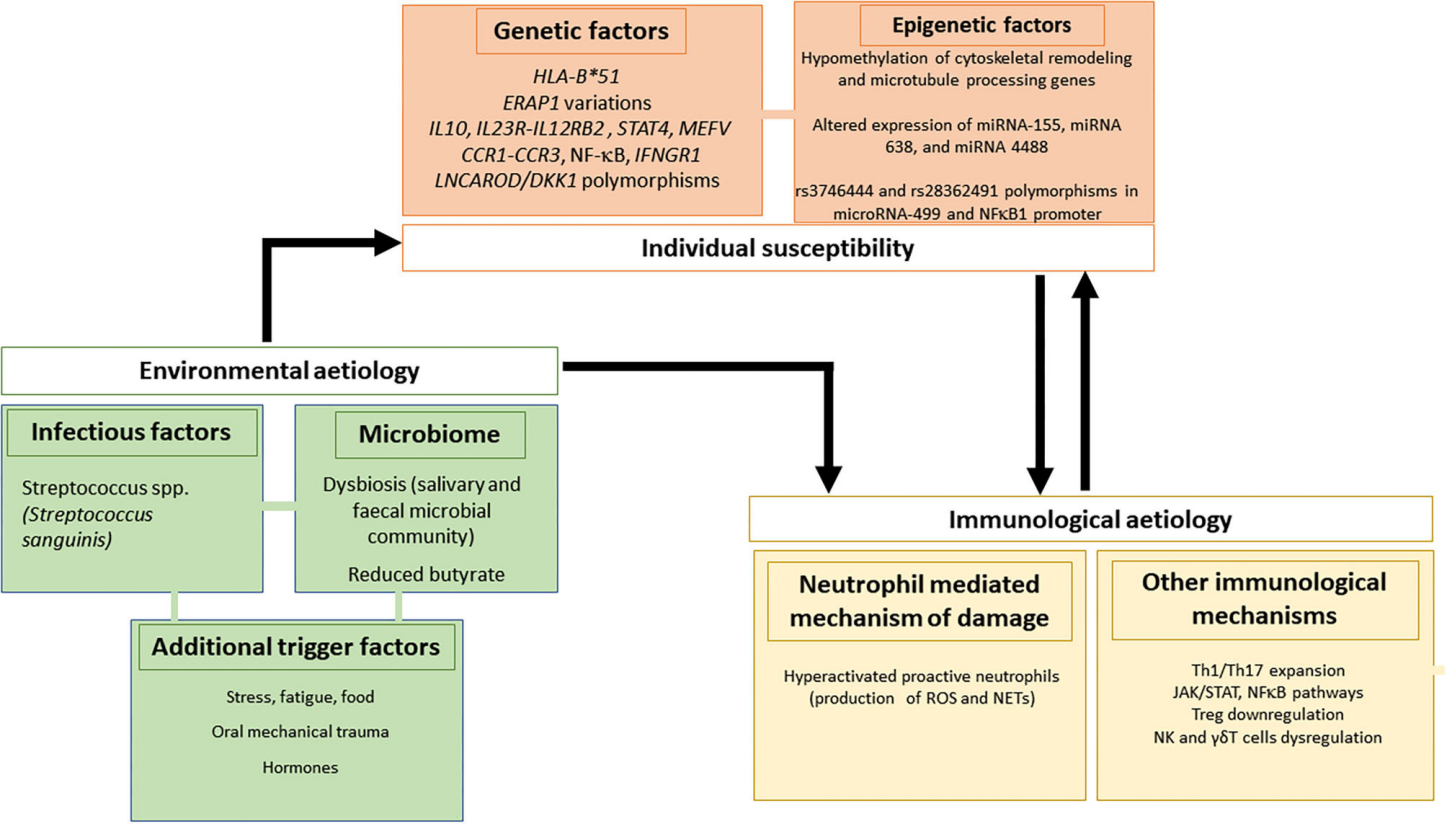
Genetic/epigenetic, infectious and immunological factors involved in the pathogenesis of Behçet's syndrome.

In addition, there is evidence suggesting a crucial role also of γδT cells as responsible for the induction and/or maintenance of the proinflammatory environment characteristic of the disease (Figure 2).

In the meanwhile, a growing interest is focusing on the introduction of new validated biomarkers for disease diagnosis. In this context, in the absence of validated laboratory parameters for BS diagnosis, an emerging role is being attributed to specific miRNA signature associated with BS. Moreover, the identification of miRNA profiles connected with specific BS features could also provide valuable insights to reveal key pathogenetic mechanism, and also representing future targets for tailored therapeutic interventions.

Considering that in BS common inflammatory biomarkers poorly reflect disease course, NETs are also emerging as promising biomarkers for monitoring disease activity and could represent a significant step forward in the clinical approach, as well as in the knowledge of BS pathogenesis.

Finally, a specific gut microbiota (GM) characterized by changes in SCFA profiles (among which especially butyrate) is now known. Intriguingly, a growing interest in nutritional interventions aimed at restoring this GM imbalance to ameliorate both pathogenic and clinical features in BS is recently arising (43, 44) (NCT03962335).

Further studies are needed to understand the role of these processes to better clarify BS etiopathogenesis.

## Author Contributions

IM, AB, and GE conceived and drafted the manuscript. GS-D and HD critically revised the manuscript. All authors contributed to the article and approved the submitted version.

## Conflict of Interest

The authors declare that the research was conducted in the absence of any commercial or financial relationships that could be construed as a potential conflict of interest.

## Publisher's Note

All claims expressed in this article are solely those of the authors and do not necessarily represent those of their affiliated organizations, or those of the publisher, the editors and the reviewers. Any product that may be evaluated in this article, or claim that may be made by its manufacturer, is not guaranteed or endorsed by the publisher.
